# Swallowing Artificial Teeth

**Published:** 1858-04

**Authors:** 


*Swallowing Artificial Teeth.*—Scarcely a month passes in which we do not hear of some one swallowing artificial teeth, and that serious consequences have not more frequently resulted from it, is really wonderful. A medical gentleman of Baltimore called on the senior editor a short time since, in a state of great alarm from having swallowed, an hour or two before, a gold plate, extending from the first molar on one side to the second bicuspis of the superior maxillary on the other, with three artificial teeth, two bicuspids and a lateral incisor, and a clasp at each extremity. His first impulse after the occurrence of the accident, was to call on one of his medical friends, who regarding the case as one of dentistry, and as not coming strictly within his province, advised him to consult the senior editor. Although the teeth and plate were now beyond the reach of the dentist, he nevertheless expressed the belief that inasmuch as they had made their way through the œsophagus into the stomach without much difficulty, they would traverse the remaining portion of the alimentary canal, and escape from the anus without injury, which they did in seventy-two hours from the time they were swallowed.

A similar accident occurred in Cincinnati, Ohio, about four years ago, and more recently, another in London. We have also heard of several other accidents of the same kind, and it is stated in a late number of the Boston Traveller, that Mr. Bartlett, of Swampscott, Mass., swallowed while asleep, a gold plate with six artificial teeth attached, which lodging in the upper part of the œsophagus, came very near causing his death, but fortunately he was relieved the next day, by the removal of the piece by Dr. Pearson of Salem.

The only fatal result from an accident of this kind of which we recollect to have ever heard, occurred some months ago at the Bellevue Hospital, in New York. The subject Mr. McDougall, having swallowed a gold plate with two artificial teeth attached, they lodged in the œsophagus about two inches above the cardiac orifice of the stomach, as was, after his death, ascertained by *post-mortem* examination of the body. They produced ulceration at the point where they had lodged, which extended through into the pericardium, and ultimately caused the death of the patient.

Dentists applying artificial teeth which are not securely attached by clasps to remaining natural teeth, should impress upon their patients the importance of removing them from the mouth at night before going to bed, and indeed this should always be done.

